# Anti‐inflammatory properties of lemon‐derived extracellular vesicles are achieved through the inhibition of ERK/NF‐κB signalling pathways

**DOI:** 10.1111/jcmm.17404

**Published:** 2022-07-04

**Authors:** Stefania Raimondo, Ornella Urzì, Serena Meraviglia, Marta Di Simone, Anna Maria Corsale, Nima Rabienezhad Ganji, Antonio Palumbo Piccionello, Giulia Polito, Elena Lo Presti, Francesco Dieli, Alice Conigliaro, Riccardo Alessandro

**Affiliations:** ^1^ Dipartimento di Biomedicina, Neuroscienze e Diagnostica Avanzata Università degli Studi di Palermo Palermo Italy; ^2^ Central Laboratory of Advanced Diagnosis and Biomedical Research (CLADIBIOR) AOUP Paolo Giaccone Palermo Italy; ^3^ Dipartimento di Scienze e Tecnologie Biologiche Chimiche e Farmaceutiche Università degli Studi di Palermo Palermo Italy; ^4^ Institute for Biomedical Research and Innovation (IRIB) National Research Council (CNR) Palermo Italy

**Keywords:** Citrus Limon L., ERK1/2, inflammatory cytokines, NF‐κB, plant‐derived extracellular vesicles

## Abstract

Chronic inflammation is associated with the occurrence of several diseases. However, the side effects of anti‐inflammatory drugs prompt the identification of new therapeutic strategies. Plant‐derived extracellular vesicles (PDEVs) are gaining increasing interest in the scientific community for their biological properties. We isolated PDEVs from the juice of Citrus limon L. (LEVs) and characterized their flavonoid, limonoid and lipid contents through reversed‐phase high‐performance liquid chromatography coupled to electrospray ionization quadrupole time‐of‐flight mass spectrometry (RP‐HPLC–ESI‐Q‐TOF‐MS). To investigate whether LEVs have a protective role on the inflammatory process, murine and primary human macrophages were pre‐treated with LEVs for 24 h and then were stimulated with lipopolysaccharide (LPS). We found that pre‐treatment with LEVs decreased gene and protein expression of pro‐inflammatory cytokines, such as IL‐6, IL1‐β and TNF‐α, and reduced the nuclear translocation and phosphorylation of NF‐κB in LPS‐stimulated murine macrophages. The inhibition of NF‐κB activation was associated with the reduction in ERK1‐2 phosphorylation. Furthermore, the ability of LEVs to decrease pro‐inflammatory cytokines and increase anti‐inflammatory molecules was confirmed ex vivo in human primary T lymphocytes. In conclusion, we demonstrated that LEVs exert anti‐inflammatory effects both in vitro and ex vivo by inhibiting the ERK1‐2/NF‐κB signalling pathway.

## 1 INTRODUCTION

Plant‐derived extracellular vesicles (PDEVs) are raising a growing interest in the scientific community since several studies have shown that they contain functional biomolecules, such as proteins, RNA, metabolites and lipids.^1^, ^2^, ^3^, ^4^ By their complex molecular content, PDEVs influence mammalian cells^5^ and therefore represent the mediators of the cross‐kingdom interaction. In particular, accumulating studies suggest that PDEVs possess anti‐cancer, anti‐inflammatory and anti‐oxidant activities, making them therapeutically attractive.^6^, ^7^, ^8^

Inflammation is a protective host response to infection and tissue damage, characterized by a series of reactions, such as vasodilation and recruitment of immune system cells to the site of infection or tissue damage.^9^ Ordinarily, inflammation is beneficial to the organism and can resolve in a relatively brief time; nevertheless, deregulated inflammatory responses can cause long‐term damage in tissues, thus contributing to the development of acute and chronic inflammatory diseases, such as ulcerative colitis, obesity, neurodegenerative diseases, diabetes type II and cancer.^10^

To date, increasing experimental evidence highlights a key role of PDEVs in promoting an anti‐inflammatory response in in vitro and in vivo models. Grape exosome‐like nanoparticles (GELNs) have a protective effect against colitis, reducing colon shortening and mortality in DSS‐treated mice.^4^, ^11^ Moreover, ginger‐derived exosome‐like nanoparticles were able to induce nuclear translocation of Nrf2 in murine macrophages and upregulate haem oxygenase 1 (HO‐1) and interleukin 10 (IL‐10) levels.^12^ Another group demonstrated that ginger‐derived nanoparticles (GDNPs) could protect mice from acute and chronic colitis by up‐regulating anti‐inflammatory cytokines, such as IL‐10 and interleukin 22 (IL‐22) and down‐regulating the pro‐inflammatory ones, like interleukin 6 (IL‐6), tumour necrosis factor α (TNF‐α) and interleukin 1 β (IL‐1β).^13^ Nanovesicles isolated from broccoli, called BDNs, decreased TNF‐α, interleukin 17A (IL‐17A) and interferon γ (IFN‐γ) in colonic tissues of mice affected by colitis and showed protective properties by acting on dendritic cells.^14^

Nuclear factor kappa‐light‐chain‐enhancer of activated B cells (NF‐κB) is a transcriptional factor that plays a key role in the regulation of inflammation since it mediates the gene induction and functions in both innate and adaptive immune cells.^15^ NF‐κB is known to activate the pro‐inflammatory response in macrophages, cells of the innate immune system that reside in tissues and are activated in the first line against infections.^16^ Lipopolysaccharide (LPS) binds the toll‐like receptor 4 (TLR4), a receptor present on macrophages, and via MyD88 and TRIF, two adaptors of TLR,^17^ induces M1 polarization and expression of pro‐inflammatory cytokines through NF‐κB activation.^18^ NF‐κB regulates in turn a large number of inflammatory genes, such as interleukin 6 (IL‐6), tumour necrosis factor α (TNF‐α), interleukin 1‐β (IL‐1β), interleukin 12p40 (IL‐12p40) and cyclooxygenase 2 (COX‐2).^16^, ^19^

T lymphocytes also play an important role in the inflammatory process, taking part in the adaptive immune response, and their activation is regulated by NF‐κB.^20^ A de‐regulation of NF‐κB in T lymphocytes may be associated with autoimmune diseases and inflammatory conditions.^21^

Another important player in inflammatory responses is the extracellular signal‐regulated kinase 1/2 (ERK1‐2), a serine/threonine‐protein kinase of the MAPKs family whose activation can occur in response to pro‐inflammatory stimuli.^22^ Moreover, the ERK1‐2 signalling pathway can activate NF‐κB and increase pro‐inflammatory cytokines.^23^

The inhibition of NF‐κB and ERK1‐2 signalling pathways by natural compounds could represent an attractive therapeutic strategy to contrast inflammatory disease.

We and other research groups have previously isolated and characterized nanovesicles from Citrus limon juice (LEVs) and investigated their anti‐cancer effects both in vitro and in vivo.^6^, ^24^ Furthermore, we have evaluated, for the first time, the effects of a natural product containing LEVs on modifiable risk factors in healthy volunteers, showing a reduction in LDL cholesterol.^25^

In this study, we carried out a more in‐depth analysis of the components of LEVs and we explored their possible protective effect against inflammatory processes in both in vitro and ex vivo inflammatory models.

## 2 MATERIALS AND METHODS

### 2.1 Lemon extracellular vesicles isolation

Lemon extracellular vesicles (LEVs) were isolated from Citrus limon L. juice as previously described.^6^ Fruits, obtained from a private farmer, were carefully washed in water and manually squeezed. The juice was sequentially centrifuged at 3000× *g* for 15 min and 10,000× *g* for 1 h. The supernatant was filtered at 0.8 and 0.45 μm pore filter and centrifuged at 16,500× *g* for 3 h. The supernatant was then ultra‐centrifuged at 100,000× *g* for 105 min in a Type 70 Ti, fixed angle rotor, and the pellet was suspended in PBS. LEVs quantification was determined with the Bradford assay (Pierce). On average, we recovered 600 μg of vesicles from 240 ml of Citrus juice.

### 2.2 Reversed‐Phase HPLC/MS experiments

Water and acetonitrile were of HPLC/MS grade. Formic acid was of analytical quality. The HPLC system was an Agilent 1260 Infinity. A reversed‐phase Agilent Poroshell 120 EC‐C18 column (50 × 3.0 mm, particle size 2.7 μm) with a Phenomenex C18 security guard column (4 × 3 mm) was used. The flow rate was 0.4 ml/min, and the column temperature was set to 30°C. The eluents were formic acid–water (0.1:99.9, v/v; phase A) and formic acid–acetonitrile (0.1:99.9, v/v; phase B). The following gradient was employed: 0–10 min, linear gradient from 5% to 95% B; 10–12 min, reconditioning to 5% B; 12–15 min, 5% B isocratic. LEVs obtained as in paragraph 2.1, were stored at −20°C before analysis. LEVs were left to warm to room temperature and directly injected without further extraction. The injection volume was 15 μl. The eluate was monitored through MS TIC. Mass spectra were obtained on an Agilent 6540 UHD accurate‐mass Q‐TOF spectrometer equipped with a Dual AJS ESI source working in negative and positive mode. N_2_ was employed as desolvation gas at 320°C and a flow rate of 7 L/min.

The nebulizer was set to 20 psig, the Sheath gas temperature was set at 295°C, and a flow of 8 L/min. A potential of 2.6 kV was used on the capillary for negative ion mode. The fragmentor was set to 175 V. MS spectra were recorded in the 100–3200 m/z range.

Eriocitrin, limonin‐17‐β‐D‐glucoside and hesperidin were used as standards. Three stock solutions containing 100 mg/L of each compound were prepared in methanol.

Then other solutions were prepared by successive dilutions with water, using stock solutions, with the following concentrations: 0.1, 1, 2.5, 5 mg/L (for eriocitrin), 0.1, 1, 2.5, 5, 10 mg/L (for hesperidin) and 0.1, 1, 2.5, 5 mg/L (for limonin‐17‐β‐D‐glucoside).

A linear relationship between peak area and concentration (1–5 mg/L) was observed with a correlation coefficient *R* ^2^ = 0.9799 and *R* ^2^ = 0.9978 respectively for eriocitrin and limonin‐17‐β‐D‐glucoside, whereas for hesperidin, it was observed a linear relationship between peak area and concentration (1–10 mg/L) with a correlation coefficient *R* ^2^ = 0.9811.

The relationship between peak areas (*y*) and concentrations in mg/L (*x*) was *y* = 452,530*x* + 153,304 for eriocitrin, *y* = 261,592*x* + 238,426 for hesperidin and *y* = 337,984*x* + 40,774 for limonin‐17‐β‐D‐glucoside. The minimum detection limit was 0.1 mg/L for all the compounds.

LEV samples for HPLC were analysed ‘as is’. Reproducibility was verified with 3 replicates.

### 2.3 Cell cultures

The murine macrophage RAW264.7 cell line was purchased from ATCC. Cells were cultured in Dulbecco's modified Eagle's medium (DMEM) supplemented with 10% foetal bovine serum (FBS, Euroclone), 2 mM L‐glutamine (Euroclone), 100 U/ml penicillin and 100 μg/ml streptomycin (Euroclone).

Peripheral blood mononuclear cells (PBMCs) were isolated from the peripheral blood of healthy donors using Ficoll gradient centrifugation (Lympholyte‐H, Euroclone). After their viability was checked by Trypan blue exclusion test at the optical microscope, PBMCs were cultured in RPMI 1640 supplemented with 15 mM HEPES Buffer, 100 IU/ml penicillin, 0.1 mg/ml streptomycin, 2 mM L‐glutamine and 10% heat‐inactivated foetal bovine serum (Euroclone).

Human monocytes were isolated and purified from PBMCs of buffy coats obtained from healthy donors. The procedures adopted in this study were in agreement with the Helsinki Declaration and were approved by the Ethics Committee of the University Hospital of Palermo, Palermo, Italy (ethical protocol code N° 03/2019 and N° 01/2022). After isolation from PBMCs, using Pan Monocyte Kit on MACS Separator (Miltenyi Biotec) according to protocol, monocytes were cultured at a concentration of 2 × 10^5^/ml in presence of macrophage colony‐stimulating factor (M‐CSF; 20 ng/ml) for 1 week to induce differentiation into macrophages.

### 2.4 Cell viability and cytotoxicity assays

Cell viability was determined by MTT assay as previously described.^6^ RAW264.7 cells were seeded in triplicate in 96‐well plates; 24 h post‐seeding, cells were treated with different doses of LEVs for 24 and 48 h. The absorbance was measured by ELISA reader at 540 nm (Microplate Reader, BioTek). Values are expressed as a percentage of cell growth versus control (untreated cells).

Cell viability was also evaluated by using RealTime‐Glo™ MT Cell Viability Assay (Catalogue number G9711, Promega). Briefly, RAW264.7 cells were plated in triplicate into white‐walled, opaque 96‐well plates; 24 h post‐seeding, cells were treated with different doses of LEVs. At the same time, the 2× MT Cell Viability Substrate and NanoLuc® Enzyme were added. The luminescent signal, which correlates with the number of metabolically active cells, was measured at 24, 48 and 72 h by Glomax (Promega).

To evaluate whether LEVs could affect the vitality of primary cells, 2.5 × 10^5^ PBMCs were cultured in a U‐bottomed 96‐well plate for 3, 24 and 48 h with or without 10 and 25 μg/ml LEVs. Cell surface staining to identify CD4^+^, CD8^+^ and TCRγδ^+^ cells was performed as described below. Subsequently, cells were washed twice in PBS, re‐suspended in 1× Binding Buffer and stained with APC‐conjugated anti‐Annexin V (BD Biosciences) for 15 min at room temperature protected from the light. Then, they were washed once with PBS and stained with 7‐AAD Staining Solution (Miltenyi Biotec) for 5 min.

For the detection of cytotoxicity, the CellTox™ Green Cytotoxicity Assay (Catalogue number G8741, Promega) was used. RAW264.7 and PBMCs were cultured in triplicate into white‐walled, opaque assay 96‐well plates; 24 h post‐seeding, cells were treated for 24 and 48 h with different doses of LEVs. Changes in membrane integrity occurring as a result of cell death were measured by the CellTox™ Green Cytotoxicity Assay following the manufactures instructions. The fluorescence, proportional to cytotoxicity, was measured by Glomax (Promega).

### 2.5 Real‐Time PCR

RAW264.7 cells were seeded in 12‐well plates at 1 × 10^5^ cells/well; 24 h post‐seeding cells were pre‐treated with 10 or 25 μg/ml of LEVs for 24 h and then were stimulated with 500 ng/ml of LPS for 6 h. Primary human macrophages were seeded in 12‐well plates; 24 h post‐seeding cells were pre‐treated with 25 μg/ml of LEVs for 24 h and then were stimulated with 100 ng/ml of LPS for 4 h. At the end of the treatments, total RNA was extracted using Illustra™ RNA spin mini‐RNA isolation Kit (GE Healthcare). The RNA was reverse transcribed to cDNA using the High‐Capacity cDNA Reverse Transcription kit (Applied Biosystems). Then, the cDNA was subjected to quantitative real‐time reverse transcriptase‐polymerase chain reaction (RT‐PCR) analysis. The sequences of the primers used were as follows:

**Table:** 

Gene	Forward	Reverse
Murine		
GAPDH	CCCAGAAGACTGTGGATGG	CAGATTGGGGGTAGGAACAC
TNF‐α	CACGTCGTAGCAAACCACCAAGTGGA	TGGGAGTAGACAAGGTACAACCC
IL‐6	GGTACATCCTCGACGGCATCT	GTGCCTCTTTGCTGCTTTCAC
IL‐1β	CAACCAACAAGTGATATTCTCCATG	GATCCAACACTCTCCAGCTGCA
Human		
GAPDH	ATGGGGAAGGTGAAGGTCG	GGGTCATTGATGGCAACAATAT
TNF‐α	CCAGGCAGTCAGATCATCTTCTC	AGCTGGTTATCTCTCAGCTCCAC
IL‐6	GGTACATCCTCGACGGCATCT	GTGCCTCTTTGCTGCTTTCAC
IL‐1β	ACAGATGAAGTGCTCCTTCCA	GTCGGAGATTCGTAGCTGGAT

Real‐time PCR was performed using Step One™ Real‐time PCR System Thermal Cycling Block (Applied Biosystem) in a 20 μl reaction containing 300 nM of each primer, 2 μl template cDNA and 18 μl 2× SYBR Green I Master Mix. The PCR was run at 95°C for 20 s followed by 40 cycles of 95°C for 3 s and 60°C for 30 s. GAPDH was used as the endogenous control. Relative changes in gene expression between control and treated samples were determined using the ΔΔCt method.

### 2.6 Enzyme‐linked immunosorbent assay (ELISA) assays

The amounts of IL‐6, TNF‐α and IL‐1β in culture supernatants were determined by using mouse IL‐6‐, TNF‐α‐ and IL‐1β‐specific ELISA kits (Thermo Fisher Scientific). RAW264.7 cells were seeded at 10 x 10^5^ cells per well in 12‐well plates; 24 h after seeding, cells were treated for 24 h with 10 or 25 μg/ml of LEVs and then exposed to LPS (500 ng/ml) for 6 h. At the end of the experimental time, the conditioned medium was collected and centrifuged to remove cellular debris. The amount of TNF‐α was also measured in the conditioned medium of human primary macrophages treated for 24 h with 25 μg/ml of LEVs and then exposed to LPS (100 ng/ml) for 4 h. The ELISA assays were then performed according to the manufacturer's instructions.

### 2.7 Western blotting

Total proteins, nuclear and cytoplasmic fractions from RAW264.7 cells treated with LEVs (25 μg/ml) and then exposed to LPS (500 ng/ml) for 30 min and 3 h were isolated and analysed by SDS‐PAGE followed by Western blotting. Nuclear and cytoplasmic fractions were obtained by using Nuclear Extract Kit (Active Motif), following the manufacturer's instructions. Antibodies used in the experiments were as follows: anti‐NF‐κB antibody (Novus), anti‐PNF‐κB (Invitrogen), anti‐ERK1/2 (Novus), anti‐PERK1/2 (Santa Cruz), anti‐Histone H1 (Active Motif) and anti‐Tubulin (Santa Cruz). The membranes were incubated with HRP‐conjugated secondary antibody (Thermo Fisher Scientific), and the chemiluminescent signal was detected by Chemidoc (Bio‐Rad).

### 2.8 Confocal microscopy

RAW264.7 cells were pre‐treated with LEVs (25 μg/ml) and then exposed to LPS (500 ng/ml) for 3 h. At the end of the experiments, the cells were fixed with 4% PFA, permeabilized with 0.1% TritonX‐100, incubated with anti‐NF‐κB antibody (Novus) for 1 h and then washed and incubated with Goat anti‐Rabbit IgG Secondary Antibody, DyLight 488 (Invitrogen) for 1 h. Nuclei were stained with Hoechst (Molecular Probes, Life Technologies). The samples were analysed by confocal microscopy (Nikon A1).

### 2.9 Phenotypical analysis

A total of 2.5 × 10^5^ PBMCs were seeded for 96 h in a U‐bottomed 96‐well plate with or without 10 and 25 μg/ml LEVs. Positive control was performed using Dynabeads CD3/CD28 T‐cell Expander (Invitrogen Life Technologies). The phenotype of CD4, CD8 and T cells was determined using Alexa Fluor405‐conjugated anti‐CD45RA, PE/Texas Red‐conjugated anti‐CD27, PerCP‐Cy5‐5‐conjugated anti‐CD4, APC‐Cy7‐conjugated anti‐CD8, PE‐conjugated anti‐TCR and FITC‐conjugated anti‐CD3 MAbs (all purchased by Miltenyi Biotec). After 20 min of staining at room temperature in the dark, cells were washed with FACS buffer (PBS supplemented with 2% FBS and 2 mM EDTA) and analysed by flow cytometry on a FACS Aria Flow Cytometer using FACS Diva software.

### 2.10 Markers analysis

Peripheral blood mononuclear cells were cultured for 3, 24 and 48 h in a U‐bottomed 96‐well plate with or without 10 and 25 μg/ml LEVs, to a density of 2.5 × 10^5^ cells/well. Activation markers of CD4^+^, CD8^+^ and TCRγδ^+^ cells (previously identified) were determined using APC‐conjugated anti‐CD69 and PE‐Cy7‐conjugated anti‐CD25 mAbs (Miltenyi Biotec). After staining, cells were washed and analysed by flow cytometry on a FACS Aria Flow Cytometer using FACS Diva software.

### 2.11 Proliferation assay

Proliferation was assessed by staining cells with 5 μM Tag‐it Violet Proliferation Cell Tracking Dye (BioLegend). After checking that all cells were Tag‐it Violet labelled, they were cultured in a U‐bottomed 96‐well plate for 6 days with or without 10 and 25 μg/ml LEVs. Cells treated with 5 μg/ml Phytohemagglutinin‐L (PHA‐L) and were used as a positive control. At the end of the incubation period, cells were collected and stained for CD4, CD8 and TCRγδ as prior mentioned and analysed by flow cytometry.

### 2.12 Cytokines expression assay

Peripheral blood mononuclear cells, pre‐treated for 24 h with 10 and 25 μg/ml LEVs, were stimulated with 1 μg/ml Ionomycin and 150 ng/ml Phorbol 12‐Myristate 13‐Acetate (PMA) for 4 h, in presence of 10 μg/ml inhibitor of trans‐Golgi function (Monensin). After the incubation, cells were stained for surface markers (CD4, CD8, CD3 and TCRγδ) as previously described. Intracellular cytokine staining was performed using Inside Stain Kit (Miltenyi Biotec) according to the manufacturer's instructions. PE‐conjugated anti‐TNF‐α, PE/Texas Red‐conjugated anti‐IL‐17A and APC‐conjugated anti‐IFN‐γ mAbs were used to stain intracellular targets. After two more washes in PBS containing 1% FCS, the cells were analysed via flow cytometer.

### 2.13 Statistical analysis

#### 2.13.1 In vitro experiments

Data are reported as mean ± standard deviation (SD) of biological replicates. Statistical analysis was performed using GraphPad Prism software (GraphPad Software, Inc.). The statistical significance of the differences was analysed using a two‐tailed Student's *t*‐test. A *p*‐value ≤0.05 was considered significant.

#### 2.13.2 Ex vivo experiments

Expression of surface and intracellular markers was determined by flow cytometry on a FACS Aria Flow Cytometer using FACS Diva software. Data were analysed on FlowJo software (Tree Star, version 10.5.3). The gating strategy involved progressively measuring total cells, viable cells only, lymphomonocytes and specific cell types. For every sample, 100,000 nucleated cells were acquired and values are expressed as a percentage of viable lymphomonocytes. Statistical significance was determined by the Kruskal–Wallis test considering *p* < 0.05 (*) as significant. Statistical analysis was conducted using GraphPad Prism software (version 8.0).

## 3 RESULTS

### 3.1 Metabolomic content of LEVs

We performed a metabolomic analysis on LEVs that identified 45 compounds (Figure S1). The identified products are summarized in Table S1, including molecular formula, chemical class, retention time (min) and experimental and calculated m/z in negative ion mode. Detected compounds can be classified into different families such as organic acids, flavonoids, limonoids, cinnamic acid derivatives, lysophospholipids, an acyl‐thioester, carbohydrate, a phenolic acid derivative and two nucleotide derivatives.

The concentrations of eriocitrin (assigned to peak **16**), hesperidin (assigned to peak **24**) and limonin‐17‐β‐D‐glucoside (assigned to peak **18**) were calculated from the experimental peak areas by interpolation to standard calibration curves.

Also, to perform a semi‐quantitative analysis, eriocitrin, hesperidin and limonin‐17‐β‐D‐glucoside were regarded as secondary standards. Their calibration curves were used to determine the content of compounds belonging to three categories: eriocitrin calibration curve for non‐methoxylated flavonoids (corresponding to peaks **6**, **7**, **9**, **12**, **13**, **17** and **21**), hesperidin calibration curve for methoxylated flavonoids (corresponding to peaks **14**, **15**, **19**, **20** and **23**) and limonin‐17‐β‐D‐glucoside calibration curve for limonoids (corresponding to peaks **25**, **26**, **27** and **32**).

Concentrations (mg/L) of compounds in LEVs and relative standard deviations were calculated as the mean of three replicates, as summarized in Table 1.

**TABLE 1:** Mean concentrations and their relative standard deviations of non‐methoxylated flavonoids, methoxylated flavonoids and limonoids in lemon‐derived extracellular vesicles (LEVs)

Compound	Mean conc. LEVs (mg/L)
Non‐methoxylated flavonoids	
Eriocitrin	3.86 ± 0.69
Eriodictyol diglucoside	0.1 ± 0.01
Quercetin‐Glu‐Rha‐Glu	<0.1
Rutin	0.13 ± 0.03
Vicenin 2	0.27 ± 0.07
Eriodictyol‐Glu‐Rha‐Glu	<0.1
Luteolin rutinoside	0.34 ± 0.11
Naringin	<0.1
Methoxylated flavonoids	
Hesperidin	2.00 ± 0.82
Diosmetin diglucoside	2.02 ± 0.94
Neohesperidin	0.79 ± 0.30
Diosmetin‐glucoside	0.58 ± 0.37
Isorhamnetin‐neohesperoside	0.35 ± 0.17
Diosmin	0.21 ± 0.18
Limonoids	
Limonin‐17‐β‐D‐glucoside	0.43 ± 0.08
Nomilinic acid‐17‐β‐D‐glucoside	0.31 ± 0.22
Nomilinic acid‐β‐glucopiranoside	0.33 ± 0.14
Obacunone glucoside	0.1 ± 0.02
Limonin	<0.1

LEVs possess a variety of flavonoids, such as eriocitrin, quercetin, vicenin‐2, naringin, hesperidin and limonoids, like limonin. Most of these natural compounds exert beneficial roles, such as anti‐inflammatory,^26^, ^27^, ^28^ anti‐oxidant,^29^, ^30^ anti‐cancer properties,^31^, ^32^, ^33^, ^34^ neuroprotective effects^35^, ^36^ and contrast obesity in mice.^37^

The identification of flavonoids content in LEVs prompted us to investigate the role of LEVs in inflammatory models.

### 3.2 LEVs decreased pro‐inflammatory cytokines levels in LPS‐stimulated macrophages

**FIGURE 1:**
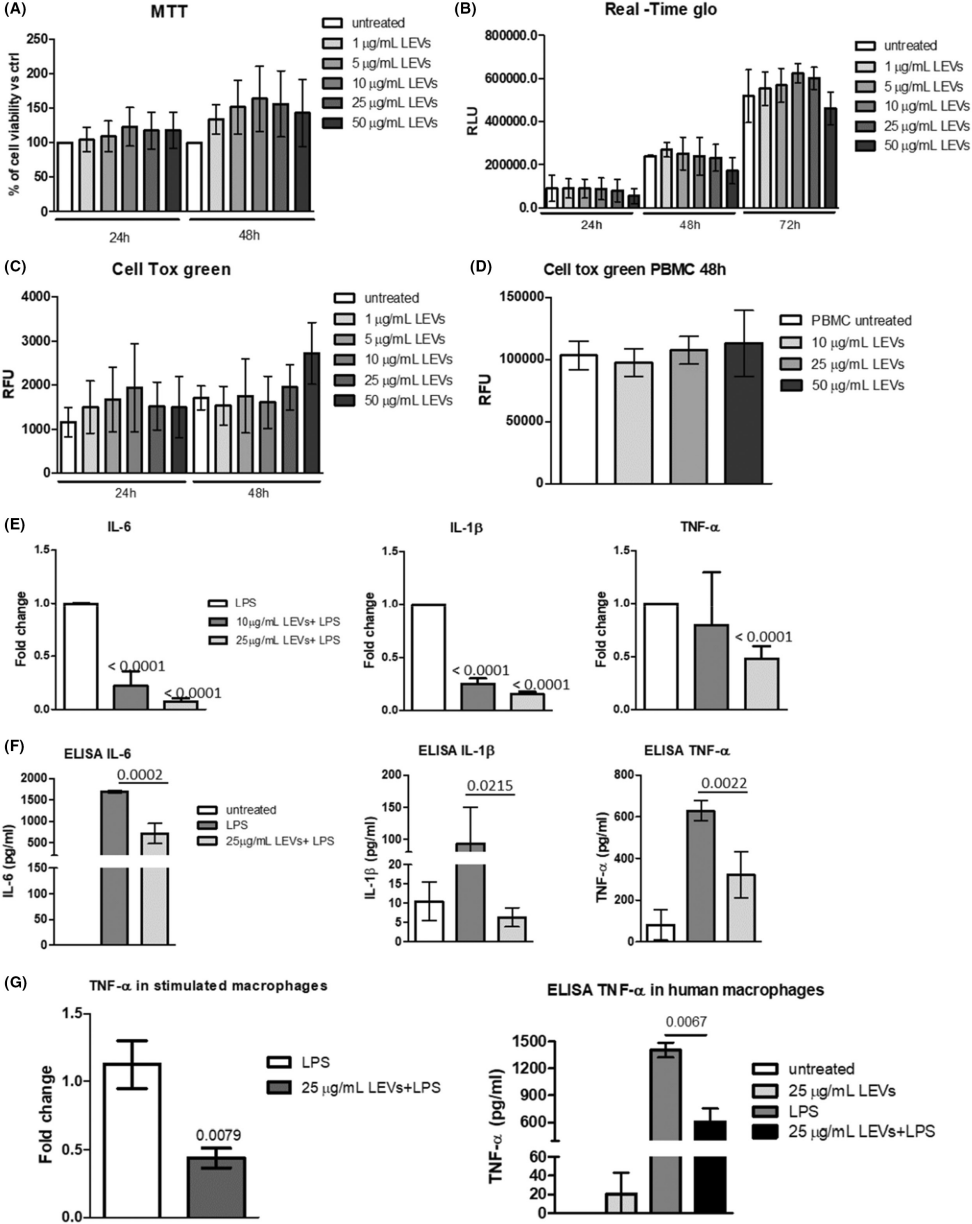
(A) MTT assay of RAW264.7 cell line treated with increasing doses of LEVs (1, 5, 10, 25 and 50 μg/ml) for 24 and 48 h. (B) RealTime‐Glo of RAW264.7 cell line treated with different doses of LEVs (1, 5, 10, 25 and 50 μg/ml) for 24, 48 and 72 h. (C) Cell Tox green assay of RAW264.7 cell line treated with different doses of LEVs (1, 5, 10, 25 and 50 μg/ml) for 24 and 48 h. (D) Cell Tox green assay of human primary PBMCs treated with different doses of LEVs (10, 25 and 50 μg/ml) for 48 h. (E) RT PCR of IL‐6, IL‐1β and TNF‐α mRNA levels in RAW264.7 cell line pre‐treated with LEVs (10 and 25 μg/ml) for 24 h and then stimulated with LPS (500 ng/ml). (F) ELISA assays of IL‐6, IL‐1β and TNF‐α protein levels in conditioned media of RAW264.7 cell line pre‐treated with LEVs (25 μg/ml) for 24 h and then stimulated with LPS (500 ng/ml). (G) RT PCR (left panel) and ELISA assay (right panel) of TNF‐α levels in human primary macrophages pre‐treated with LEVs (25 μg/ml) for 24 h and then stimulated with LPS (100 ng/ml)

Firstly, to test the safety of LEVs we performed three assays, the MTT assay, RealTime Glo and Cell Tox green, aimed at assessing both cell viability and cell death by using different doses of LEVs. As shown in Figures 1A,B, LEVs did not significantly affect murine macrophages' cell viability and did not induce cytotoxicity (Figure 1C) at all tested doses. We observed a slight reduction in viability and increase in cytotoxicity, although not statistically significant, with the highest dose (50 μg/ml); therefore, this dose was not considered for subsequent experiments. These in vitro results were supported by ex vivo experiments; we observed that LEVs did not have a cytotoxic effect on human PMBCs (Figure 1D).

To investigate whether LEVs had a protective effect on inflammation, we pre‐treated RAW264.7 for 24 h with LEVs and then induced inflammatory stimulus with LPS (500 ng/ml), a commonly used inducer, both in vitro and in vivo. Then, the gene expression and protein levels of three pro‐inflammatory cytokines: IL‐6, IL‐1β and TNF‐α were analysed. We observed that pre‐treatment with LEVs significantly downregulated the gene expression levels of these cytokines compared with LPS‐stimulated cells in a dose‐dependent manner (Figure 1E). Moreover, as shown in Figure 1F, these results were confirmed at the protein level through ELISA assays; in fact, LPS treatment induced the release of pro‐inflammatory cytokine by murine macrophages, while LEVs (25 μg/ml) pre‐treatment contrasted LPS‐induced IL‐6, IL‐1β and TNF‐α levels in the conditioned media of cells (Figure 1F). The protective effects of LEVs observed in vitro were further confirmed ex vivo, using human macrophages. We demonstrated that pre‐treatment with LEVs inhibited TNF‐α production at both gene and protein levels compared with the LPS‐stimulated cells (Figure 1G), while we did not observe any modulation of IL‐6 and IL‐1β (data not shown).

### 3.3 LEVs achieved anti‐inflammatory properties through the inhibition of the ERK1/2‐NF‐κB signalling pathway

Once we have demonstrated the ability of LEVs to counteract LPS‐induced pro‐inflammatory cytokine production, we investigated the underlying signalling. NF‐κB is considered the master regulator of the inflammatory response, it is known that its activation promotes the gene expression of IL‐6, IL‐1β and TNF‐α.^15^

**FIGURE 2:**
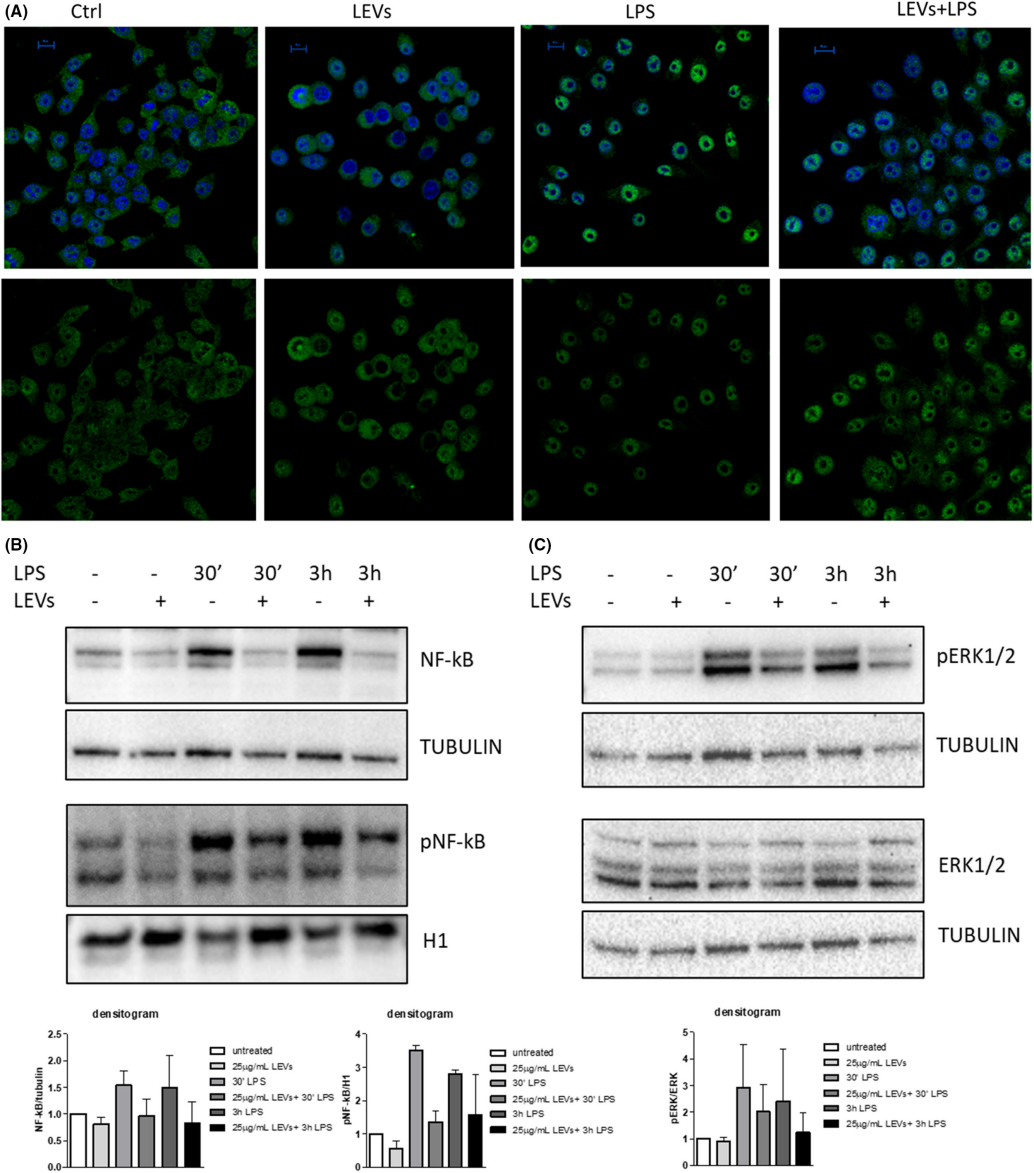
(A) Confocal analysis of NF‐κB (green) in RAW264.7 cell line pre‐treated with LEVs (25 μg/ml) for 24 h and then stimulated with LPS (500 ng/ml) for 3 h, nuclear counterstaining was performed using Hoescht (blue). The nuclear focal plane is reported in the figure (scale bar = 10 μm). (B) Western Blot analysis of NF‐κB, Tubulin, pNF‐κB and H1 in RAW264.7 cell line pre‐treated with LEVs (25 μg/ml) for 24 h and then stimulated with LPS (500 ng/ml) for 30 min and 3 h. (C) Western Blot analysis of pERK1‐2, ERK1‐2 and Tubulin in RAW 264.7 cell line pre‐treated with LEVs (25 μg/ml) for 24 h and then stimulated with LPS (500 ng/ml) for 30 min and 3 h

We studied the localization of NF‐κB using confocal microscopy. As shown in Figure 2 A, in murine macrophages stimulated with LPS for 3 h the localization of NF‐κB was predominantly nuclear, however, pre‐treatment with LEVs reduced the nuclear translocation of NF‐κB, whose signal was lower in the nuclei of pre‐treated cells than in LPS‐stimulated ones. To support this result with quantitative data, we evaluated the protein levels of NF‐κB in the total protein lysates and of its phosphorylated form (pNF‐κB) in the nuclear fraction lysates, through Western blot analysis. We observed that the treatment with LPS increased NF‐κB levels and pNF‐κB nuclear localization after 30 min and 3 h as expected from literature data, while the pre‐treatment with LEVs inhibited the effects induced by LPS (Figure 2B and Figure S2).

It is known that the MAPKs cascade is involved in the inflammatory response; in particular, several natural compounds showed the ability to inhibit the ERK signalling pathway^38^; we, therefore, analysed whether LEVs were able to contrast inflammation using this mechanism. As shown in Figure 2C (and Figure S3), LPS increased the protein levels of pERK1‐2 after 30 min 3 h, while the pre‐treatment with LEVs was able to counteract this effect. Overall, these results demonstrated for the first time the anti‐inflammatory properties of LEVs in LPS‐stimulated immune cells. These effects are partially achieved by the inhibition of the ERK1‐2/NF‐κB signalling pathways.

### 3.4 Effect of LEVs on phenotypic changes and activation of CD4, CD8 and γδ T cells

To extend our result to other effectors of inflammatory responses, we analysed whether LEVs can induce activation of acquired immune cells such as CD4, CD8 and γδ T cells through the analysis of CD69 and CD25 membrane expression.

**FIGURE 3:**
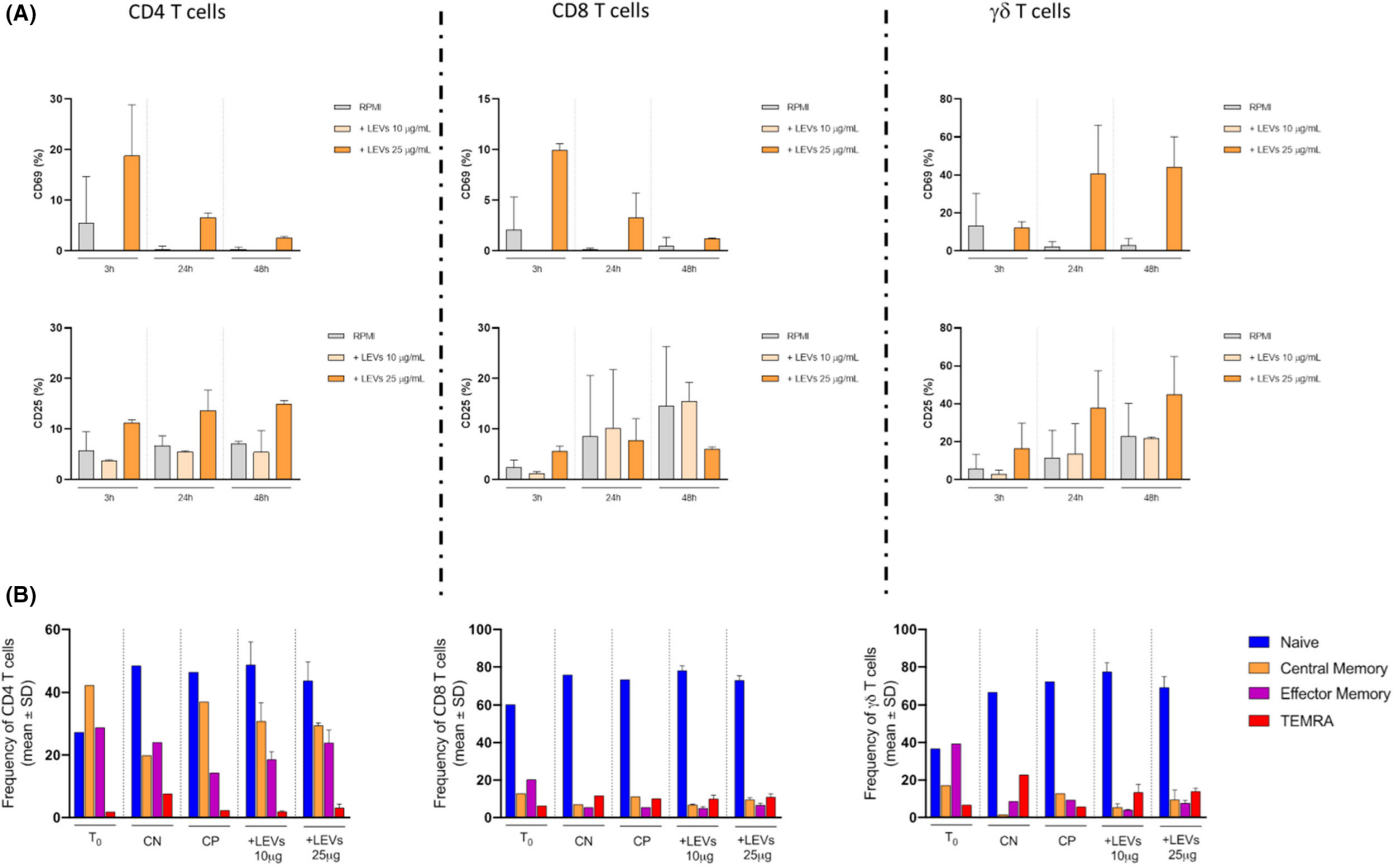
(A) Activation markers (CD69 and CD25) expression on CD4, CD8 and γδ T cells in presence of LEVs at three different time points, 3 h‐24 h‐48 h. The grey bar represents the untreated condition, while light and dark orange bars represent treated conditions with 10 and 25 μg/ml of LEVs, respectively. (B) The phenotype of CD4, CD8 and γδ T cell subsets was evaluated after 4 O.N, with or w/o 10 and 25 μg/ml of LEVs. Stimulation of PBMCs with anti‐CD3/CD28 beads was used as the positive control. The percentage of naive, central memory, effector memory and T_EMRA_ subsets was determined based on CD45RA and CD27 differential expression

The flow cytometric analysis showed that CD4 and CD8 T cells, when treated with 25 μg/ml LEVs, increased earlier marker expression CD69 at all experimental time points compared with control, showing a simultaneous progressive reduction trend over time. CD25 expression was more homogeneous either in the absence or presence of LEVs, at different concentrations and at different coculture times, showing a little increase when lemon vesicles are used at 25 μg/ml. Regarding γδ T cells, we noticed an increase in CD69 expression with 25 μg/ml LEVs only at 24 and 48 h compared with unstimulated conditions; unlike CD4 and CD8, this subset instead showed an increase in expression overtime at this concentration. As for the late activation marker expression CD25, γδ T cells were influenced only by 25 μg/ml LEVs and exhibited a similar increase as compared to control and from 3 to 48 h (Figure 3A).

CD4, CD8 and γδ T cells were stained for CD27 and CD45RA to identify their differentiation phenotype by flow cytometry. Results showed that LEVs, after 4 days, did not induce any differentiation in the cells analysed, both at 10 and 25 μg/ml (Figure 3B).

**FIGURE 4:**
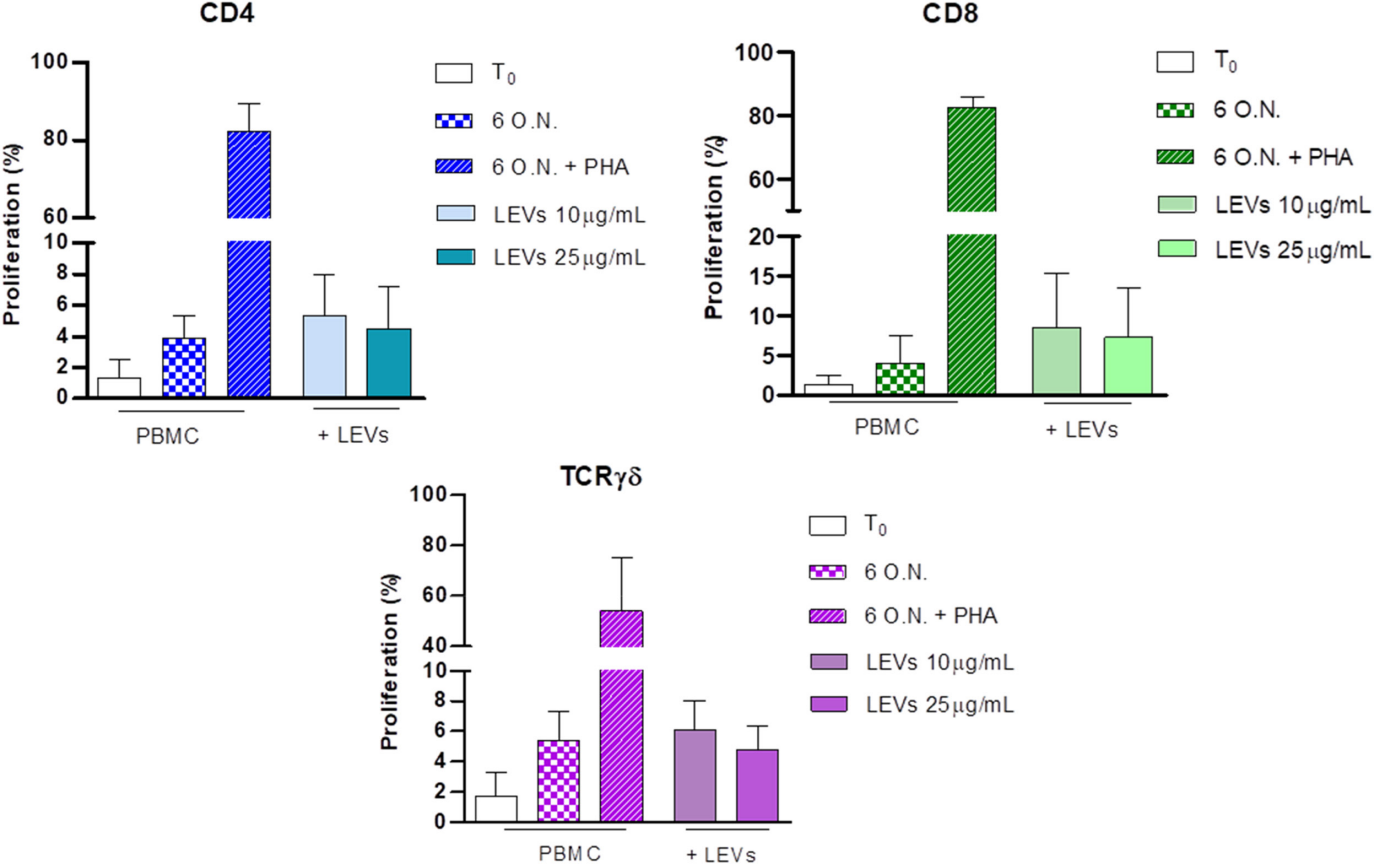
Influence of LEVs on CD4, CD8 and γδ T cell proliferation was evaluated after 6 days at two different concentrations, 10 and 25 μg/ml. PBMCs stimulated with PHA were used as the positive control (striped bar). The percentage of proliferated cells was determined using Tag‐it Violet

Moreover, we evaluated the ability of LEVs to induce proliferation of CD4, CD8 and γδ T cells; after 6 days, no changes were observed at both concentrations compared with spontaneous proliferation (Figure 4).

### 3.5 Modulation of apoptosis by LEVs on CD4, CD8 and γδ T cells

**FIGURE 5:**
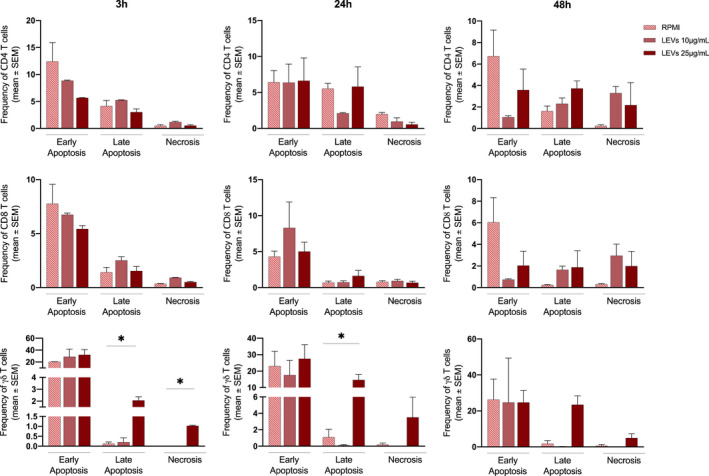
Apoptosis assay was performed on CD4+, CD8+ and γδ T cells treated with 10 and 25 μg/ml of LEVs for 3, 24 and 48 h. Early apoptosis, late apoptosis and necrosis was determined using Annexin V and 7AAD markers

Furthermore, we analysed the influence of LEVs on early and late stages of apoptosis together with necrosis by flow cytometry. The analysis, obtained by annexin V and PI staining, showed that CD4 and CD8 T cells were not affected by LEVs coculture (Figure 4), while we observed for γδ T cells a concentration‐dependent LEVs‐induced late apoptosis and necrosis (Figure 5).

### 3.6 LEVs impaired pro‐inflammatory activity and stimulated anti‐inflammatory properties

**FIGURE 6:**
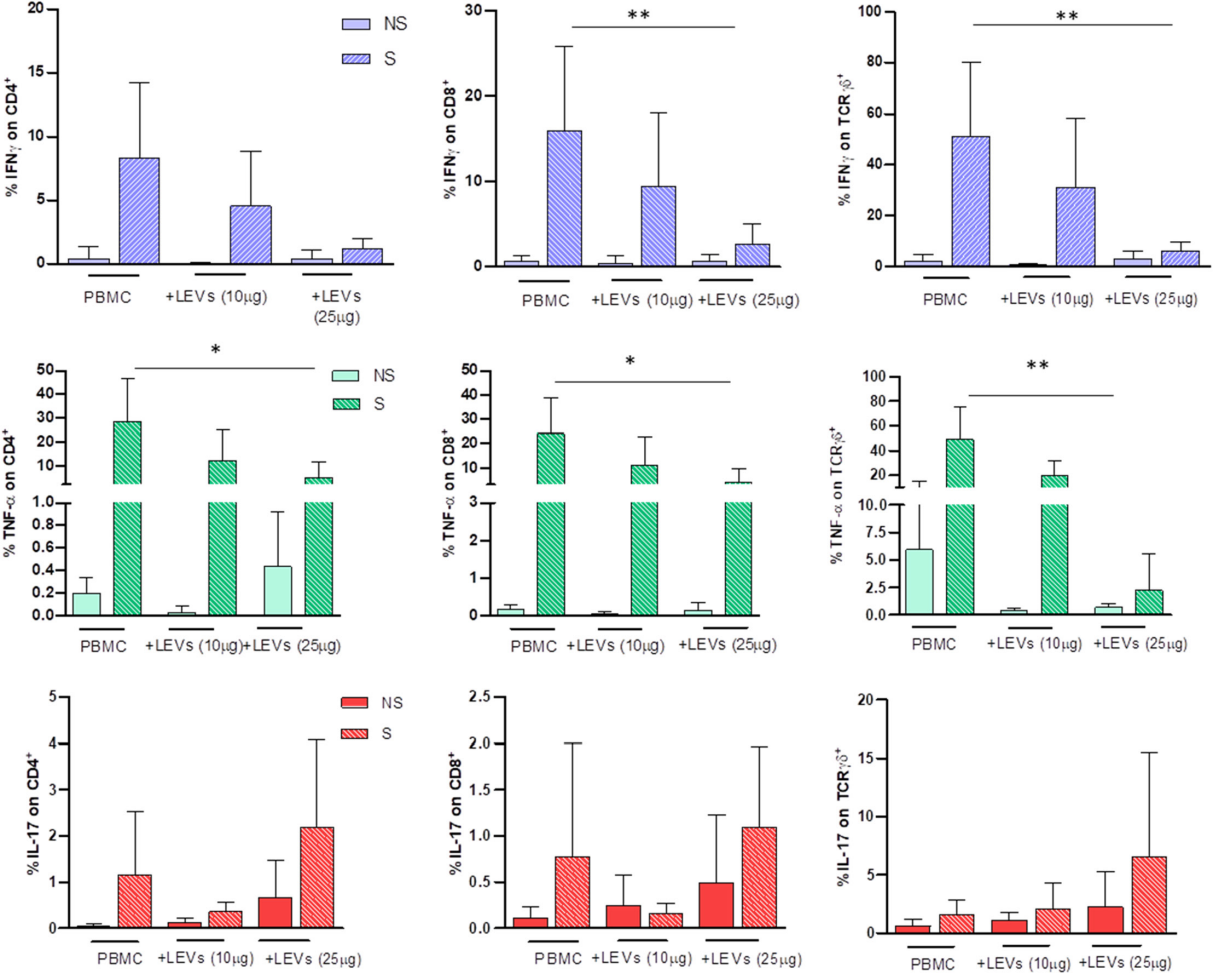
Pro‐inflammatory cytokine expression on CD4, CD8 and γδ T cells was evaluated by flow cytometry. The effect of 10 and 25 μg/ml of LEVs was compared with untreated PBMC. Every condition was performed with (S, striped bar) or w/o (NS, full/coloured bar) ionomycin and PMA. Statistical significance was determined by Kruskal–Wallis. *p* < 0.05 (*), *p* < 0.01 (**)

Finally, we analysed the functional activity of CD4, CD8 and γδ T cells upon coculture with LEVs in terms of production of pro‐inflammatory and anti‐inflammatory cytokines to understand whether these vesicles can modulate the inflammatory functions of the primary immune cells. Of note, when pre‐treated with LEVs for 24 h at 10 and 25 μg/ml, CD4, CD8 and γδ T cells, upon short‐term stimulation with ionomycin and PMA, showed a significantly reduced expression of IFN‐γ and TNF‐α in a dose‐dependent manner. The analysis of IL‐17 production showed a different behaviour with an increase in production upon coculture with LEVs at 25 mg/ml, even though the percentage was low reaching 5% maximum on γδ T cells (Figure 6).

**FIGURE 7:**
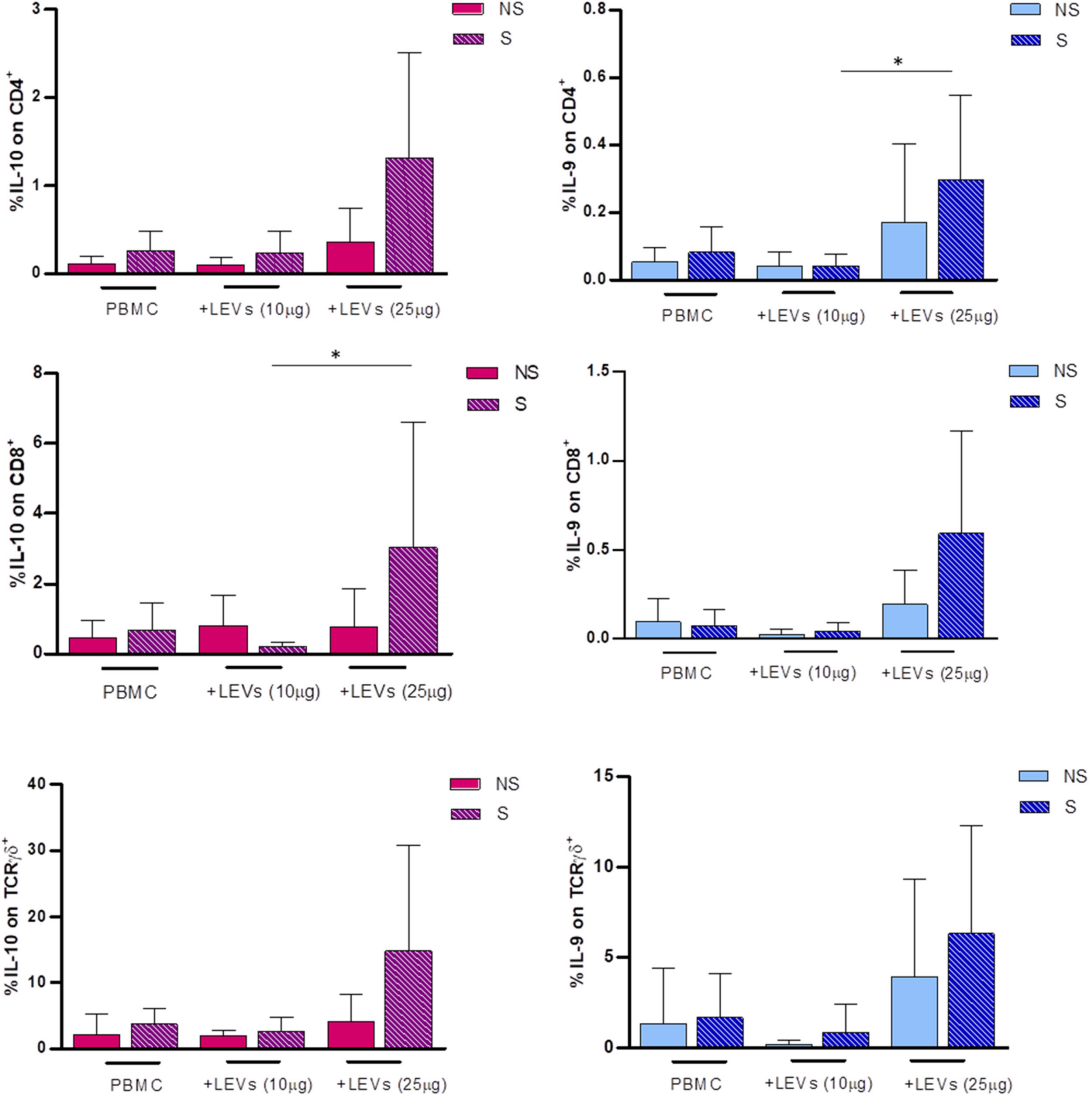
Anti‐inflammatory cytokine expression on CD4, CD8 and γδ T cells was evaluated by flow cytometry. The effect of 10 and 25 μg/ml of LEVs was compared with untreated PBMC. Every condition was performed with (S, striped bar) or w/o (NS, full/coloured bar) ionomycin and PMA. Statistical significance was determined by Kruskal–Wallis. *p* < 0.05 (*), *p* < 0.01 (**)

In parallel, an increase in IL‐10 and IL‐9 expression, in the presence of 25 μg/ml LEVs, for all three subsets was highlighted, confirming their anti‐inflammatory effect (Figure 7).

## 4 DISCUSSION

The possibility of using natural compounds as anti‐inflammatory molecules is stimulating a strong interest among the scientific community since the existing therapies have several and toxic side effects.^10^ However, the clinical use of natural bioactive compounds is limited due to their poor chemical stability and bioavailability^39^; extraction processes, as well as storage conditions,^40^ may lead to deterioration of the chemical compounds^41^ that may also result in the production of harmful metabolites.^42^

In the last years, PDEVs are raising interest among researchers, since they showed the ability to interact with mammalian cells and exert attractive biological properties, such as anti‐cancer and immune‐modulatory activities.^5^ Many studies have already demonstrated that PDEVs possess anti‐inflammatory properties; in particular, those isolated from ginger, grape and broccoli.^4^, ^11^, ^14^ In this study, we analysed the anti‐inflammatory properties of EVs isolated from lemon juice using in vitro and ex vivo models.

Once isolated EVs from Citrus limon juice, we characterized flavonoids, limonoids and lipids, and we found that among the identified compounds, LEVs contain hesperidin and eriocitrin, two flavonoids with well‐recognized beneficial properties.^27^, ^30^, ^31^ The great interest in their use as anti‐inflammatory agents is paralleled to studies aimed at encapsulating these natural compounds in lipid structures to increase their stability and availability.^43^, ^44^, ^45^ Since EVs are naturally occurring nanoparticles, they may represent an advantage in transporting biomolecules to target cells.^46^ Moreover, our data indicated that LEVs contain several flavonoids that simultaneously interact with target cells; their synergic or additive actions define EV biological properties.

Several phospholipid species were present in LEVs, in agreement with what is reported in the literature,^4^, ^47^, ^48^, ^49^ this can partially explain the beneficial effects of LEVs. In particular, we identified lyso‐phosphatidylinositol (lyso PI), lyso‐phosphatidylethanolamine (lysoPE), lyso‐phosphatidylcholine (lysoPC) and lyso‐phosphatidylserine (lysoPS). Phosphatidylcholine (PC) was also found in grapefruit,^50^ grape^4^ and ginger^47^—derived EVs. In 2018, Teng et al.^47^ demonstrated that PC presence was essential for the internalization of ginger‐EVs by intestinal Ruminococcaceae; moreover, PC improved the migration of orally administrated EVs from the intestine to the liver in vivo. Phosphatidylethanolamine (PE) was described in grape‐EVs^4^ and in nanovesicles from *Craterostigma plantagineum* and *Zingiberis rhizoma*.^48^

Once we examined LEVs content, we tested their effects on the proliferation of normal target cells. The safety of LEVs is in accordance with findings of other research groups; in fact, while PDEVs from different species demonstrated the ability to inhibit cancer cell growth, they do not affect normal cell proliferation^51^, ^52^; this is one of the most attractive characteristics of PDEVs making them possible preventive compounds and safe vehicles for drug delivery.

Inflammation is the leading cause of several diseases and both natural compounds^10^, ^53^ and EVs isolated from different plant sources^12^, ^13^, ^14^ showed anti‐inflammatory abilities. The majority of the findings regard the anti‐inflammatory effects of PDEVs on a murine model of colitis.^4^, ^11^, ^14^

Here, we found that treatment with LEVs counteracts the inflammatory stimulus induced by LPS in both in vitro and ex vivo models. Our results are in line with what has been recently observed for PDEVs from cabbage; in this study, the authors found that the PDEVs prevent inflammation in LPS‐stimulated murine macrophages through the inhibition of IL‐1β and IL‐6 gene expression and protein levels.^54^

In the current study, for the first time, we correlated LEV‐mediated decreases in pro‐inflammatory cytokines with the inhibition of ERK1‐2 and the NF‐κB pathway. NF‐κB is one of the master regulators of the inflammatory pathway and its activation promotes the transcription of several pro‐inflammatory genes, such as IL‐6, TNF‐α, IL‐1β, IL‐12p40 and COX‐2.^19^ Therefore, targeting the NF‐κB pathway may provide benefits in controlling inflammatory stimuli. Polyphenols regulate NF‐κB activation, exerting anti‐inflammatory properties.^55^, ^56^ We found that LEVs were able to decrease the total amount of NF‐κB protein, its phosphorylation and nuclear localization. The ability of LEVs to decrease NF‐κB levels is associated with the reduction in pro‐inflammatory cytokines, and it is shared with many flavonoids and other natural compounds.^57^

In addition to the above‐mentioned pathway, we found that ERK phosphorylation decreased in LEV‐treated cells; this result was consistent with findings that demonstrated that several natural compounds, like docosahexaenoic acid and quercetin, exert anti‐inflammatory properties by the inhibition of ERK1‐2 phosphorylation.^38^, ^58^, ^59^ Overall, the ability of LEVs to simultaneously inhibit multiple inflammatory pathways, like NF‐κB and ERK1‐2, encourages further studies on inflammatory disease models.

Finally, to our knowledge, this is the first study in which the effects of PDEVs on the adaptive immune system, in particular on different populations of T lymphocytes, have been investigated. The results obtained ex vivo agreed with the in vitro data; in particular, we demonstrated that LEVs did not alter the phenotype of three populations of primary T lymphocytes (CD4, CD8 and γδ); these results demonstrated the safety and biocompatibility of LEVs, thus encouraging further clinical applications. In addition, LEVs downregulated the levels of pro‐inflammatory cytokines such as IFN‐γ and TNF‐α and up‐regulated anti‐inflammatory molecules, IL‐10 and IL‐9, confirming their possible protective effect against inflammatory processes. In light of this, the development of compounds based on nanovesicles may represent an approach for the treatment of deregulated inflammatory processes in patients suffering from acute and chronic inflammatory diseases.

## 5 CONCLUSION

Overall, these data showed that the extracellular vesicles isolated from *Citrus limon* juice exhibited encouraging anti‐inflammatory properties both in vitro and ex vivo. Moreover, we identified the NF‐κB‐/ERK1‐2 signalling pathways among those affected by LEVs. The anti‐inflammatory effects of LEVs may be explained by the presence of different compounds such as flavonoids and limonoids which act synergistically and are packaged into a lipid bilayer that make them stable from degradation and easy to be absorbed by target cells. The results of this study encourage the development of novel nutraceutical products, containing LEVs, for the prevention of inflammatory diseases.

## AUTHOR CONTRIBUTIONS

**Stefania Raimondo:** Conceptualization (equal); data curation (equal); formal analysis (equal); investigation (equal); writing – original draft (equal); writing – review and editing (equal). **Ornella Urzì:** Data curation (equal); investigation (equal); methodology (equal); writing – original draft (equal). **Serena Meraviglia:** Conceptualization (equal); data curation (equal); investigation (equal); supervision (equal); writing – review and editing (equal). **Marta Di Simone:** Data curation (equal); investigation (equal); methodology (equal); writing – original draft (equal). **Anna Maria Corsale:** Data curation (equal); formal analysis (equal). **Nima Rabienezhad Ganji:** Methodology (equal). **Antonio Palumbo Piccionello:** Data curation (equal); formal analysis (equal); writing – original draft (equal). **Giulia Polito:** Methodology (equal). **Elena Lo Presti:** Data curation (equal); methodology (equal). **Francesco Dieli:** Writing – review and editing (equal). **Alice Conigliaro:** Investigation (equal); writing – review and editing (equal). **Riccardo Alessandro:** Conceptualization (equal); funding acquisition (lead); project administration (equal); supervision (equal); writing – review and editing (equal).

## CONFLICT OF INTEREST

The authors confirm that there are no conflicts of interest.

## Supporting information


Appendix S1

## Data Availability

Data are available from the corresponding authors upon reasonable request.
