# Supplementary material for: Anti‐inflammatory properties of lemon‐derived extracellular vesicles are achieved through the inhibition of ERK/NF‐κB signalling pathways
Source: J Cell Mol Med. 2022 Jul 4;26(15):4195–209. doi: 10.1111/jcmm.17404 (PMC9344827; doi:10.1111/jcmm.17404)
Supplement: Supplementary file 1 — Appendix S1 [file JCMM-26-4195-s001.pdf]

Supplementary Material

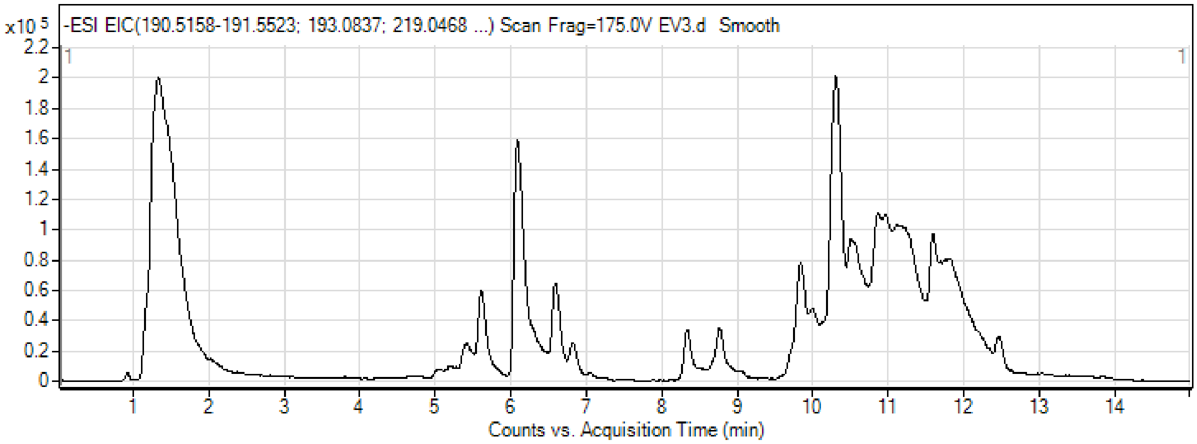

Supplementary Figure S1. Representative HPLC/ESI/QTOF trace of lemon-derived extracellular vesicles (negative ion mode).

	Compound	Molecular formula	Chemical class	ESI$^-$ [M-H]$^-$ (m/z) (*Teor.*)	ESI$^-$ [M-H]$^-$ (m/z) (*Exp.*)	Rt (min)
1	Disaccaride	$C_{12}H_{22}O_{11}$	Carbohydrate	341.1089	341.1025	1.03
2	Quinic acid	$C_7H_{12}O_6$	Organic acid	191.0561	191.0523	1.05
3	isocitric Acid	$C_6H_8O_7$	Organic acid	191.0197	191.0158	1.19
4	Citric Acid	$C_6H_8O_7$	Organic acid	191.0197	191.0158	1.51
5	(R)-(Homo)2-citrate	$C_8H_{12}O_7$	Organic acid	219.0510	219.0468	3.11
6	Eriodictyol diglucoside	$C_{27}H_{32}O_{16}$	Non-methoxylated flavonoid	611.1618	611.1541	4.51
7	Quercetin-Glu-Rha-Glu	$C_{33}H_{40}O_{21}$	Non-methoxylated flavonoid	771.1989	771.1901	4.61
8	Coumaric acid glucoside	$C_{15}H_{18}O_8$	Phenolic acid derivative	325.0929	325.0871	4.63
9	Rutin	$C_{27}H_{30}O_{16}$	Non-methoxylated flavonoid	609.1461	609.1385	4.73
10	Feruloylglucose	$C_{16}H_{20}O_9$	Cinnamic acid derivative	355.1035	355.0979	4.84
11	Sinapoylglucose	$C_{17}H_{22}O_{10}$	Cinnamic acid derivative	385.1140	385.1087	4.89

12	Vicenin 2	$C_{27}H_{30}O_{15}$	Non-methoxylated flavonoid	593.1512	593.1446	4.95
13	Eriodictyol-Glu-Rha-Glu	$C_{33}H_{42}O_{20}$	Non-methoxylated flavonoid	757.2197	757.2101	5.03
14	Diosmetin diglucoside	$C_{28}H_{32}O_{16}$	Methoxylated flavonoid	623.1618	623.1551	5.11
15	Neohesperidin	$C_{27}H_{30}O_{16}$	Methoxylated flavonoid	609.1461	609.1384	5.50
16	Eriocitrin	$C_{27}H_{32}O_{15}$	Non-methoxylated flavonoid	595.1668	595.1610	5.55
17	Luteolin rutinoside	$C_{27}H_{30}O_{15}$	Non-methoxylated flavonoid	593.1512	593.1432	5.61
18	Limonin-17-β-D-glucoside	$C_{32}H_{42}O_{14}$	Limonoid	649.2502	649.2433	5.68
19	Diosmetin-glucoside	$C_{22}H_{22}O_{11}$	Methoxylated flavonoid	461.1089	461.1036	5.79
20	Isorhamnetin-neohesperoside	$C_{28}H_{32}O_{16}$	Methoxylated flavonoid	623.1618	623.1551	5.88
21	Naringin	$C_{27}H_{32}O_{14}$	Non-methoxylated	579.1719	579.1657	5.90

			flavonoid			
22	Chlorophyll c	$C_{35}H_{30}N_4O_5$	Pigment	585.2143	585.2126	5.96
23	Diosmin	$C_{28}H_{32}O_{15}$	Methoxylated flavonoid	607.1668	607.1605	6.02
24	Hesperidin	$C_{28}H_{34}O_{15}$	Methoxylated flavonoid	609.1825	609.1763	6.12
25	Nomilinic acid-17-β-D-glucoside	$C_{34}H_{48}O_{16}$	Limonoid	711.2870	711.2806	6.35
26	Nomilinic acid-β-glucopiranoside	$C_{34}H_{46}O_{15}$	Limonoid	693.2764	693.2689	6.39
27	Obacunone glucoside	$C_{32}H_{42}O_{13}$	Limonoid	633.2553	633.2484	6.60
28	Methoxyeugenol	$C_{11}H_{14}O_3$	Methoxyphenols	193.0870	193.0837	6.78
29	Palmitoyl-CoA (ammonium salt)	$C_{37}H_{75}N_{10}O_{17}P_3S$	Acyl-thioester	1055.4172	1055.4299	8.00
30	Myristoyl-myristoleyl-sn-glycero-3-diphosphocytidine potassium salt	$C_{40}H_{71}KN_3O_{15}P_2$	Nucleotide derivative	915.3819 (M-H_2O)	915.3611 (M-H_2O)	8.16
31	1,2-Dimyristoyl-sn-glycero-3-diphosphocytidine potassium salt	$C_{40}H_{73}KN_3O_{15}P_2$	Nucleotide derivative	935.4081	935.3895	8.38
32	Limonin	$C_{26}H_{30}O_8$	Limonoid	515.1923	515.1855	8.57

				(M+FA-H)	(M+FA-H)	
33	LysoPI(8:3)	$C_{27}H_{47}O_{12}P$	Lysophospholipid	593.2732	593.2662	9.36
34	lysoPE(18:3)	$C_{23}H_{42}NO_7P$	Lysophospholipid	474.2626	474.2565	9.48
35	lysoPC(18:3)	$C_{26}H_{48}NO_7P$	Lysophospholipid	562.3150 (M+FA-H)	562.3076 (M+FA-H)	9.55
36	lysoPE(18:3) isomer	$C_{23}H_{42}NO_7P$	Lysophospholipid	474.2626	474.2568	9.61
37	lysoPC(18:3) isomer	$C_{26}H_{48}NO_7P$	Lysophospholipid	562.3150 (M+FA-H)	562.3076 (M+FA-H)	9.70
38	lysoPS(18:2)	$C_{24}H_{44}NO_9P$	Lysophospholipid	520.2681	520.2608	9.92
39	lysoPE(18:2)	$C_{23}H_{44}NO_7P$	Lysophospholipid	476.2783	476.2720	9.96
40	lysoPC(18:2)	$C_{26}H_{50}NO_7P$	Lysophospholipid	564.3307 (M+FA-H)	564.3228 (M+FA-H)	10.06
41	lysoPE(18:2) isomer	$C_{23}H_{44}NO_7P$	Lysophospholipid	476.2783	476.2725	10.11
42	lysoPC(18:2) isomer	$C_{26}H_{50}NO_7P$	Lysophospholipid	564.3307 (M+FA-H)	564.3232 (M+FA-H)	10.22
43	lysoPE(16:0)	$C_{21}H_{44}NO_7P$	Lysophospholipid	452.2783	452.2729	10.52
44	lysoPI(16:0)	$C_{25}H_{49}O_{12}P$	Lysophospholipid	571.2889	571.2824	10.56
45	lysoPC(16:0)	$C_{24}H_{50}NO_7P$	Lysophospholipid	540.3307 (M+FA-H)	540.3246 (M+FA-H)	10.67

Supplementary Table 1. Composition of lemon-derived extracellular vesicles (LEVs).

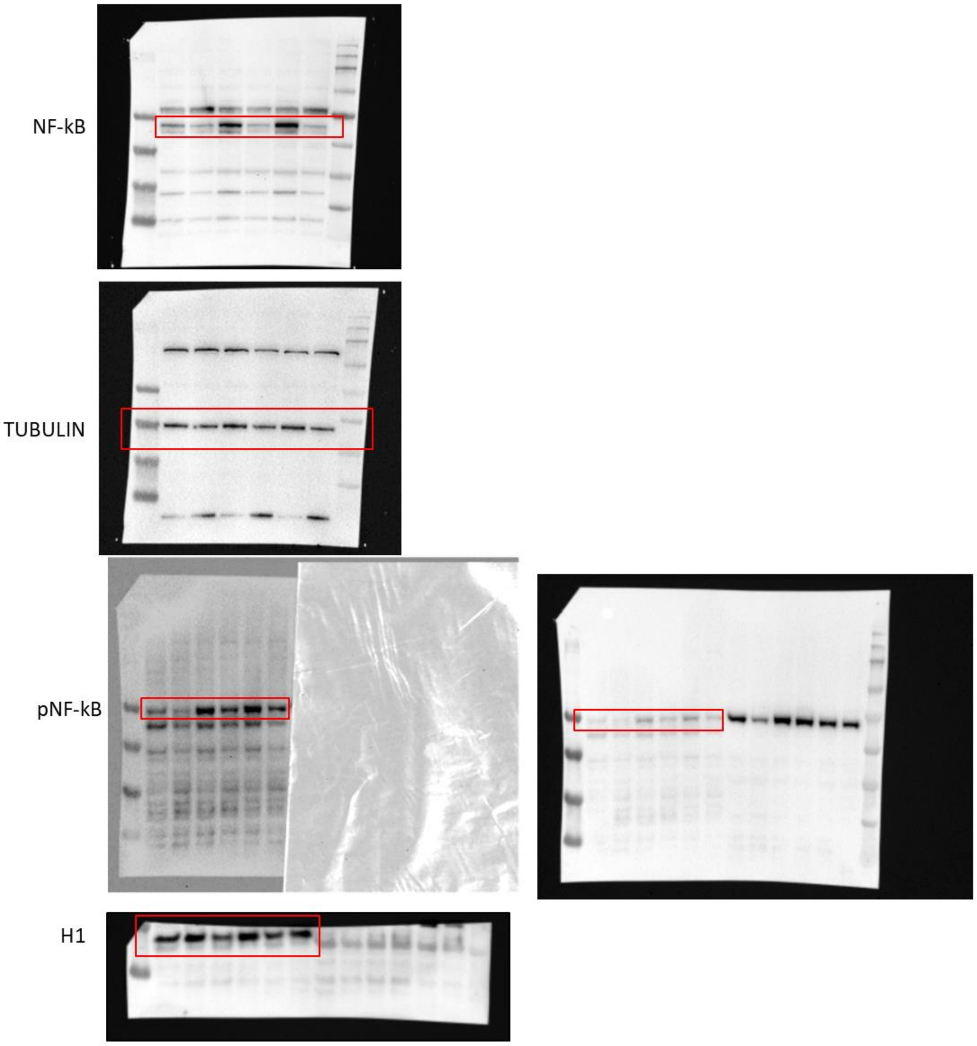

Supplementary Figure S2. Un-cropped blots of figure 4B

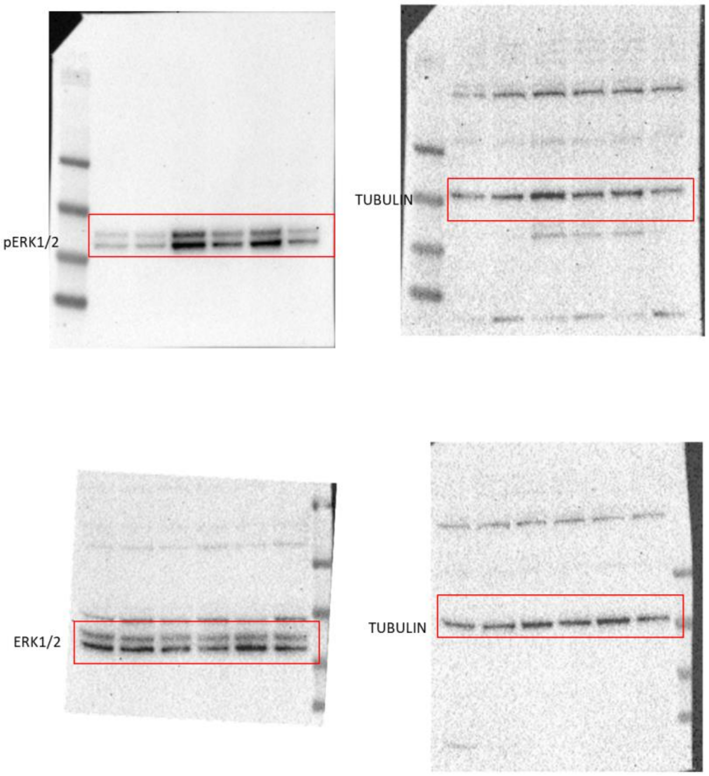

Supplementary Figure S3. Un-cropped blots of figure 4C
